# Low-Voltage Area Ablation in Addition to Pulmonary Vein Isolation in Patients with Atrial Fibrillation: A Systematic Review and Meta-Analysis

**DOI:** 10.3390/jcm13154541

**Published:** 2024-08-03

**Authors:** Stefano Valcher, Alessandro Villaschi, Giulio Falasconi, Mauro Chiarito, Filippo Giunti, Laura Novelli, Lucio Addeo, Antonio Taormina, Cristina Panico, Pietro Francia, Andrea Saglietto, Guido Del Monaco, Alessia Chiara Latini, Sebastiano Carli, Stefano Frittella, Alessandro Giaj Levra, Giulia Antonelli, Alberto Preda, Fabrizio Guarracini, Patrizio Mazzone, Antonio Berruezo, Massimo Tritto, Gianluigi Condorelli, Diego Penela

**Affiliations:** 1 Department of Biomedical Sciences, Humanitas University, 20072 Pieve Emanuele, Italy; stefano.valcher@humanitas.it (S.V.); alessandro.villaschi@humanitas.it (A.V.); mauro.chiarito@hunimed.eu (M.C.); filippo.giunti@humanitas.it (F.G.); laura.novelli@humanitas.it (L.N.); cristina.panico@hunimed.eu (C.P.); guido.delmonaco@humanitas.it (G.D.M.); alessia.latini@humanitas.it (A.C.L.); sebastiano.carli@humanitas.it (S.C.); stefano.frittella@humanitas.it (S.F.); alessandro.giajlevra@humanitas.it (A.G.L.); giulia.antonelli@humanitas.it (G.A.); massimo.tritto@materdomini.it (M.T.); gianluigi.condorelli@hunimed.eu (G.C.); 2 Olv Hospital, 9300 Aalst, Belgium; addeolucio@gmail.com; 3 Department of Medicine, Karolinska Institutet, 17177 Solna, Sweden; 4 IRCCS Humanitas Research Hospital, 20089 Rozzano, Italy; antonio.taormina@humanitas.it (A.T.); diego.penelamaceda@humanitas.it (D.P.); 5 Teknon Medical Center, 08022 Barcelona, Spain; pietro.francia@uniroma1.it (P.F.); antonio.berruezo@quironsalud.es (A.B.); 6 Campus Clínic, University of Barcelona, 08036 Barcelona, Spain; 7 Advanced Biomedical Sciences, University of Naples Federico II, 80138 Naples, Italy; 8 Department of Clinical and Molecular Medicine, Sapienza University of Rome, 00185 Rome, Italy; 9 Department of Medical Sciences, University of Turin, 10124 Turin, Italy; 10 Electrophysiology Unit, De Gasperis Cardio Center, ASST Great Metropolitan Niguarda, 20162 Milan, Italy; alberto.preda@ospedaleniguarda.it (A.P.); fabrizio.guarracini@ospedaleniguarda.it (F.G.); patrizio.mazzone@ospedaleniguarda.it (P.M.)

**Keywords:** atrial fibrillation, catheter ablation, paroxysmal atrial fibrillation, persistent atrial fibrillation, low-voltage area, metanalysis

## Abstract

**Background:** Low-voltage area (LVA) ablation, in addition to pulmonary vein isolation (PVI), has been proposed as a new strategy in patients with atrial fibrillation (AF), but clinical trials have shown conflicting results. We performed a systematic review and meta-analysis to assess the impact of LVA ablation in patient undergoing AF ablation (PROSPERO-registered CRD42024537696). **Methods:** Randomized clinical trials investigating the role of LVA ablation in addition to PVI in patients with AF were searched on PubMed, Embase, and the Cochrane Library from inception to 22 April 2024. Primary outcome was atrial arrhythmia recurrence after the first AF ablation procedure. Secondary endpoints included procedure time, fluoroscopy time, and procedure-related complication rate. Sensitivity analysis including only patients with LVA demonstration at mapping and multiple subgroups analyses were also performed. **Results:** 1547 patients from 7 studies were included. LVA ablation in addition to PVI reduced atrial arrhythmia recurrence (odds ratio [OR] 0.65, 95% confidence interval [CI] 0.52–0.81, *p* < 0.001) with a number needed to treat to prevent recurrence of 10. No difference in procedure time (mean difference [MD] −5.32 min, 95% CI −19.01–8.46 min, *p* = 0.45), fluoroscopy time (MD −1.10 min, 95% CI −2.48–0.28 min, *p* = 0.12) and complication rate (OR 0.81, 95% CI 0.40–1.61, *p* = 0.54) was observed. Consistent results were demonstrated when considering only patients with LVA during mapping and in prespecified subgroups for AF type (paroxysmal vs. persistent), multicentric vs. monocentric trial, and ablation strategy in control group. **Conclusions**: In patients with AF, ablation of LVAs in addition to PVI reduces atrial arrhythmia recurrence without a significant increase in procedure time, fluoroscopy time, or complication rate.

## 1. Introduction

Atrial fibrillation (AF) is the most common arrhythmia worldwide [1] and is associated with a high risk of mortality and morbidity [2,3]. Since the discovery of the crucial role of pulmonary veins, as triggers inducing AF [4], their isolation has become the cornerstone of AF ablation [5]. Over the years, advancements in catheters, mapping systems, and ablative techniques have significantly enhanced the efficacy of this procedure. Nevertheless, in certain patients, particularly those diagnosed with persistent AF (PeAF), the recurrence rate of arrhythmia remains notably high [6,7]. Despite various additional approaches like ablation lines, posterior wall box, and complex fractionated atrial electrogram (CFAE) ablation, none has amassed sufficient evidence to establish superiority over a pulmonary vein isolation (PVI)-only approach [8,9,10]. According to the latest guidelines a IIb class of recommendation is given to any other ablation beyond pulmonary vein isolation [11]. Low-voltage areas (LVAs) are the expression of atrial remodeling and can act as the arrhythmogenic substrate required for perpetuating AF [12]. The presence of LVAs during left atrial electroanatomical mapping (EAM) is inversely correlated with the likelihood of maintaining sinus rhythm after ablation [13]. Recently, LVAs has been proposed as a new target for ablation with promising initial results. However, targeting LVA for ablation has yielded conflicting results in the randomized clinical trials (RCTs) [14,15,16,17,18,19,20]. Previous meta-analyses have been published on the subject, but they predominantly included non-randomized trials [12,21]; moreover, in the recent years several RCTs on this topic have been added to the previous published evidence.

The aim of this systematic review and meta-analysis is to analyze only RCTs in order to provide a comprehensive synthesis of the impact of LVAs ablation in patient undergoing AF ablation (PROSPERO-registered CRD42024537696).

## 2. Methods

### 2.1. Data Sources and Search Strategy

The presented study is a systematic review and meta-analyses and was conducted in accordance with the PRISMA (Preferred Reporting Items for Systematic Review and Meta-Analysis) guidelines. PRISMA checklist was reported in the Supplementary Materials. Pubmed, Embase, and Cochrane databases were searched for relevant articles from inception to 22 April 2024 using the following keywords: “AF”, “atrial fibrillation”, “persistent atrial fibrillation”, ”paroxysmal atrial fibrillation”, “catheter ablation”, “ablation”, “pulmonary vein isolation”, “radiofrequency ablation”, “voltage area”, “voltage”, “substrate”, “fibrosis”, “fibrotic area”, “modification”, “low voltage area”, “low voltage”, “clinical trial”. No language restriction was applied. The study protocol was registered in PROSPERO (CRD42024537696).

### 2.2. Study Selection, Data Extraction and Quality Assessment

Articles retrieved from the systematic search were screened for eligibility by two independent reviewers (S.V. and L.N.) based on title, abstract and study design. All randomized studies comparing atrial arrhythmia-free survival in the group undergoing PVI plus low voltage-guided ablation (LVA ablation) versus PVI alone or in combination with other non-low voltage-guided ablation techniques (non-LVA ablation) were included. Trials with more than two groups for which a subset of interventions satisfied the inclusion criteria were kept in the analysis after having discarded the groups that did not satisfy the inclusion criteria. Studies enrolling patients with paroxysmal AF (pAF) and PeAF were included. Two authors (S.V, F.G.) independently extracted data regarding study design, population characteristics, outcomes, and follow-up, using a standardized data extraction form. Whether raw data regarding arrhythmia recurrence were not readily available in the full text article or in the Supplementary Material, extraction from the Kaplan-Meier plots was performed using the method described by Liu et al. [22]. The risk of bias was assessed independently by the same two investigators using the Cochrane Risk of Bias Tool (ROB2) for randomized studies. Five domains were assessed: (1) randomization process, (2) deviations from intended interventions, (3) missing outcome data, (4) measurement of the outcome, and (5) selection of the reported results. Conflicts in study selection, quality assessment and data extraction were discussed and resolved with a senior investigator (D.P.). In the case of studies with overlapping populations, the report with the longest follow up was selected.

### 2.3. Outcome Definition

The primary efficacy endpoint was atrial arrhythmia recurrence after the first AF ablation procedure. Secondary endpoints included procedure time, fluoroscopy time, and procedural complications.

### 2.4. Statistical Analysis

Results were pooled using a random-effects model; odds ratios (OR) for binary outcomes, and difference in means (MD) for continuous outcomes, their respective 95% confidence intervals (CI) and two-sided *p*-values for each outcome were calculated via the restricted maximum likelihood (REML) method. Hypothesis of statistical heterogeneity was tested by means of Cochran Q statistic and I^2^ values. I^2^ values of less than 25%, 25–50%, or more than 50% indicated low, moderate, or considerable heterogeneity, respectively. Prespecified sensitivity analyses for the primary outcome were performed (1) with the use of a fixed-effects model and (2) including only patients with demonstration and ablation of LVA in the experimental arm. Prespecified leave-one-out sensitivity analyses were performed for primary and secondary outcomes, iteratively removing one study at a time. Subgroup analyses based on study design, AF type, ablation strategy in the control arm, and risk of bias were also performed for the primary endpoint. Post-hoc subgroup analyses based on AF type and ablation strategy in control arm were done also for the secondary outcomes. An interaction term between subgroup and effect estimates was calculated using a REML random-effects meta-regression analysis. The number of patients needed to treat to prevent one event was calculated from weighted estimates of pooled ORs from the random effects meta-analytic model in case of significant risk difference among patients treated with LVA ablation and control group. Random-effects meta-regression analyses with the REML method were also performed to assess the presence of interaction between each of the following potential effect modifiers and treatment effect for the primary endpoint: sex, age, left ventricular ejection fraction (LVEF), left atrium diameter (LAD), CHA₂DS₂-VASc score, AF duration before ablation. Publication bias was assessed via visual inspection of funnel plots; no formal publication bias test was performed due to the low number of studies included. Statistical significance was set at *p*-value < 0.05 (two-sided). Statistical analyses were performed according to the intention-to-treat principle and using STATA (version 18; StataCorp., College Station, TX, USA).

## 3. Results

### 3.1. Search Results and Baseline Characteristics

**Figure 1:**
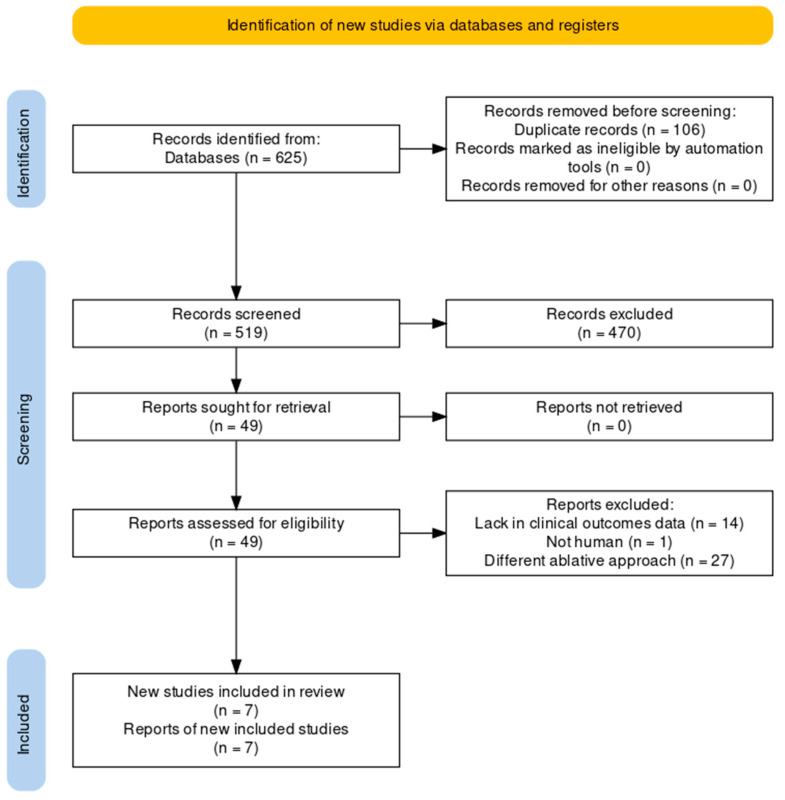
PRISMA flow-chart.

A total of 625 results were retrieved from database search. After duplicate removal and study screening, seven randomized clinical trials and a total of 1547 patients were included in the metanalysis (Figure 1). The main characteristics of the studies included are summarized in Table 1. Three out of seven studies have a single center study design. Two of these included only patients with pAF [14,15], four enrolled only patients with PeAF [16,17,18,19], and one included both populations [20]. STABLE-SR III [15] was conducted only on patients aged between 65 and 80 years. In STABLE-SR II [17], STABLE-SR III [15], ERASE [19] and VOLCANO [14] patients in the control arm underwent PVI-only ablation, while in the remaining studies, patients underwent other ablation strategies (e.g., lines, box) in addition to PVI. In the study by Kircher et al. [18], patients with pAF underwent PVI-only ablation, while those with PeAF underwent PVI plus additional ablations. In the VOLCANO [14] trial randomization occurred after voltage mapping only for patients in whom LVA were documented; only these patients were considered for the analyses. All studies utilized radiofrequency as ablative energy, while in VOLCANO [14] trial cryoballoon ablation was also performed. The follow-up data from the VOLCANO [14] trial were extracted from the latest paper published by the authors, which reports the results at two years [23].

Table 2 summarizes the characteristics of the patients included in the meta-analysis. Among 1547 patients included, 962 (62.2%) were male and 264 (17.1%) patients had pAF. LVA were found during substrate mapping in 574 (37%) patients.

### 3.2. Risk of Bias

Risk of bias for the primary endpoint was deemed to be low for four studies, whereas the other three studies presented some concerns. (Supplementary Figure S1) No publication bias was detected from the analysis of the funnel plot (Supplementary Figure S2).

### 3.3. Primary Outcome

**Figure 2:**
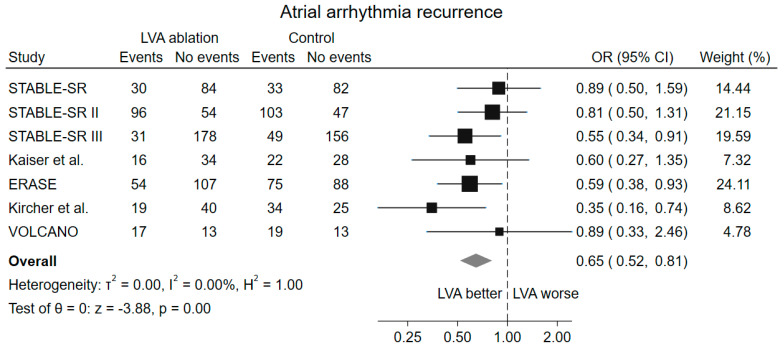
Forest plot of atrial arrhythmia recurrence. LVA low-voltage area; OR odds ratio, CI confidence interval [15,16,17,18,19,20,23].

A significant reduction in atrial arrhythmia recurrence was observed in the group randomized to LVA ablation, if present, in addition to PVI compared to the group randomized to PVI alone (OR 0.65, 95% CI 0.52–0.81, *p* < 0.001) (Figure 2). No heterogeneity among the included studies was observed (I^2^ = 0%).

The sensitivity analysis with fixed-effect model and the leave-one-out analysis for the primary outcome showed consistent findings with the main analysis (Supplementary Figure S3). The number of patients needed to treat to prevent a recurrence of atrial arrhythmia in patients undergoing LVA ablation was 10.

**Figure 3:**
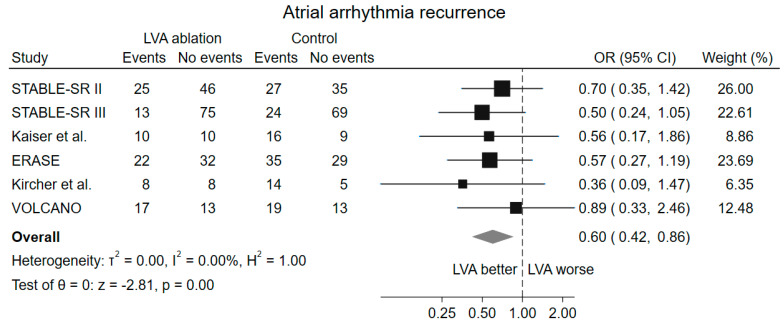
Forrest plot of atrial arrhythmia recurrence considering only the LVA population. LVA low-voltage areas, OR odds ratio, CI confidence interval [15,17,18,19,20,23].

The sensitivity analysis considering only the population of patients who had LVA on voltage mapping confirmed the reduction in atrial arrhythmia recurrence in the population randomized to low voltage ablation (OR 0.60, 95% CI 0.42–0.86, *p* = 0.005) (Figure 3). This analysis was only possible to perform on 6 out of the 7 included studies; data extraction from the STABLE-SR [16] trial was not feasible, because the authors did not report the data.

### 3.4. Secondary Outcomes

**Figure 4:**
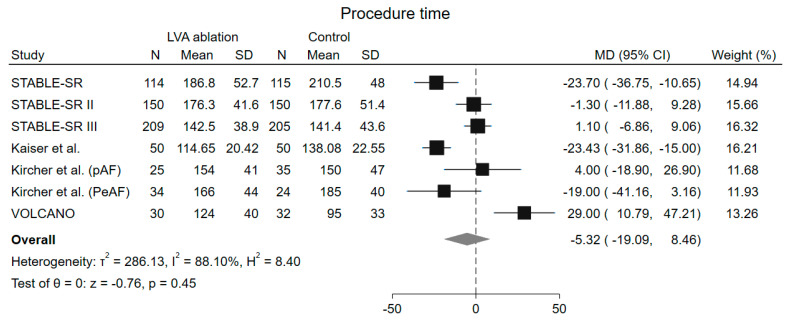
Forrest plot of procedure time. LVA low-voltages area, MD mean difference, CI confidence interval, N number of patients, SD standard deviation [15,16,17,18,20,23].

No difference in total procedure time was detected among groups (MD −5.32 min, 95% CI −19.01–8.46 min, *p* = 0.45), but a high grade of heterogeneity was observed (I^2^ = 88.10%) (Figure 4). Leave-one-out analysis demonstrated consistent results with the main analysis (Supplementary Figure S4). The ERASE [19] trial was excluded from the analysis because they reported the median instead of the mean procedure time.

**Figure 5:**
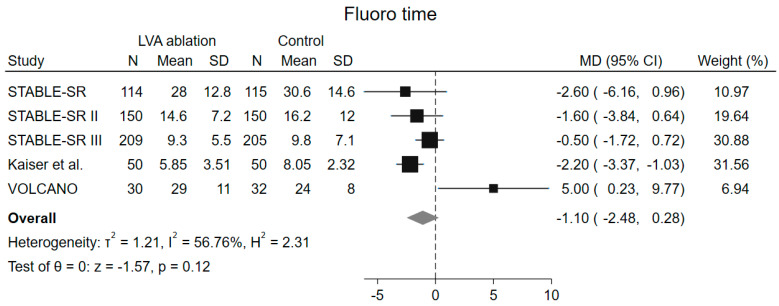
Forrest plot of fluoroscopy time. LVA low-voltages area, MD mean difference, CI confidence interval, N number of patients, SD standard deviation [15,16,17,18,23].

Fluoroscopy time was also similar among groups (MD −1.10 min, 95% CI −2.48–0.28 min, *p* = 0.12), but with a high heterogeneity among included studies (I^2^ = 56.8%). (Figure 5) At leave-one-out analysis, a significant reduction of fluoroscopy time was observed in the LVA arm after removal of the VOLCANO trial [14] (MD −1.51 min, 95% CI −2.60–−0.42, *p* = 0.007). (Supplementary Figure S5) From this analysis two studies were excluded, the ERASE [19] trial because the authors did not report the data, and the study by Kircher et al. [20] because they reported the median instead of the mean.

**Figure 6:**
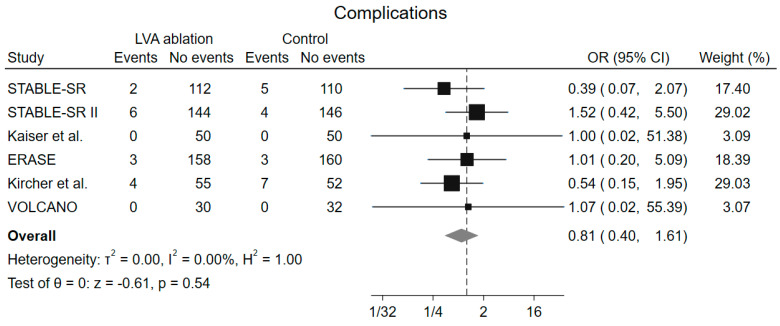
Forrest plot of complications. LVA low-voltages area, OR odds ratio, CI confidence interval [16,17,18,19,20,23].

No difference in the complication rate (OR 0.81, 95% CI 0.40–1.61, *p* = 0.54) was found among groups, and no heterogeneity was observed (I^2^ = 0%) (Figure 6). Leave-one-out analysis showed consistent result with the main analysis (Supplementary Figure S6).

### 3.5. Subgroup and Metaregression Analysis

Results from subgroup analyses (PAF vs. PeAF; Multicentric vs. Monocentric, PVI alone vs. PVI + other ablative strategies) for the primary outcome demonstrated consistent results with the main analysis and did not show any difference among subgroups (*p* > 0.05). (Supplementary Figures S7–S9) An additional subgroup analysis based on the estimated risk of bias, found no difference in the occurrence of the primary outcome among subgroups (*p* > 0.05). (Supplementary Figure S10)

Regarding secondary endpoints, subgroup analyses demonstrated a significant reduction in procedure time only among patients with PeAF (PeAF, MD −16.56 min, 95% CI −28.28–−4.84 min vs. pAF, MD 10.55 min, 95% CI −7.00–28.11 min, *p* = 0.01) and among those enrolled in studies where the control group underwent PVI + other ablative strategies (PVI+, MD −23.09 min, 95% CI −29.84–−16.34 min vs. PVI only, MD 6.79, 95% CI −6.03–19.61 min), with a significant interaction between groups (*p* < 0.001). (Supplementary Figures S11 and S12).

Meta-regression analyses did not demonstrate any significant impact of sex, age, LVEF, LAD, and CHA₂DS₂-VASc score on the effect estimate for the primary outcome (all *p* > 0.10), whereas a significant impact on the risk of the primary outcome was found for AF duration before ablation on the primary outcome (coeff. −0.02, 95% CI −0.32–−0.001, *p*= 0.043) (Supplementary Table S1).

## 4. Discussion

The main findings of this meta-analysis of RCTs are: (i) lower recurrence rate of atrial arrhythmias was found in patients assigned to LVA ablation in addition to PVI; (ii) consistent results were found in subgroup and sensitivity analyses evaluating, among the others, the type of AF, ablation strategy in the control arm and actual presence of LVA; (iii) no difference in procedure tima, fluoroscopy time, as well as in complication rate, was found among groups.

### 4.1. Presence of Low Voltages Areas

To accurately interpret the results of this meta-analysis, it is crucial to acknowledge the clinical heterogeneity among the included studies. A significant aspect is the variability in the randomization in relation to EAM. As a matter of fact, only the VOLCANO trial [14] conducted randomization after EAM, specifically selecting patients with LVAs in the LA. In contrast, the remaining studies conducted the randomization before performing the EAM. The prevalence of LVA ranged from 38 to 48% across the studies. Consequently, most of the included patients in absence of LVA have received standard PVI despite randomization. This inconsistency could potentially influence and dilute the observed effect of LVA ablation, as a significant portion of patients may not receive the benefits associated with the intervention group. For example, the ERASE trial [19] reports different atrial arrhythmia-free survival outcomes in patients without LVA across the two study arms. Specifically, in the intervention arm, atrial arrhythmia recurrences are lower in patients without LVAs compared to those in the control group. To better address this issue, we conducted a subgroup analysis of atrial arrhythmia recurrence rates specifically among patients in whom LVA were identified in the EAM. This analysis confirmed that the observed benefit in the overall population stemmed from the superiority of the LVA ablation approach.

### 4.2. Paroxysmal and Persistent Atrial Fibrillation

It is well known that the type of AF affects the outcomes of ablation procedures. In cases of PAF, maintaining sinus rhythm at a mean 12-month follow-up after PVI exceeds 80% [24], while this success rate significantly drops for PeAF [6,7], indicating a more substantial role of arrhythmogenic substrate in the arrhythmia’s maintenance [25]. Consequently, the likelihood of a positive response to LVA ablation may vary depending on the AF category.

The included RCTs enrolled patients with different types of AF. The VOLCANO and STABLE-SR III trials [14,15] focused exclusively on patients with PAF, while Kircher et al. [20] included both PAF and PeAF patients. The remaining studies enrolled PeAF patients [16,17,18,19]. To address this variability, we performed a subgroup analysis and found that the reduction in atrial arrhythmia recurrence seems consistent regardless of the AF type. Interestingly, a significant number of PAF patients exhibited LVA. This observation suggests that defining AF type solely based on the duration of arrhythmia episodes may not be sufficient for guiding the appropriate ablation strategy. Instead, a patient-specific approach that considers the presence of LVA may be more effective.

### 4.3. Relevance of the Ablation Strategy

Another relevant aspect for correctly interpreting the results of this meta-analysis is the ablation approach used in the control group. In four of the included studies (STABLE-SR II, STABLE-SR III, ERASE, and VOLCANO trials) [14,15,17,19], the control group underwent a PVI-only approach. In contrast, additional ablation strategies were permitted in the remaining studies [16,18,20]. The impact of these additional lesions on the maintenance of sinus rhythm is not clearly defined. Furthermore, there is a potential pro-arrhythmic effect due to incomplete, non-transmural lines or reconnections, which must be considered. However, the results of the subgroup analysis remained consistent with the main findings, irrespective of the ablation strategy used in the control arm. These results strengthen our findings, indicating that the benefit of LVA was not limited to studies comparing this strategy to PVI-only.

However, there was variation among studies in the approach to ablating LVAs. While some utilized homogenization of the LVAs, others targeting the LVAs through ablation lines. Determining the most effective strategy for LVA ablation extends beyond the scope of this meta-analysis and should be addressed in future research endeavors.

### 4.4. Procedure and Fluoroscopy Time

This meta-analysis also demonstrates no significant difference in procedure times in the population undergoing LVA ablation, although this result could be attributable to the patients with PeAF: in these patients additional time-consuming ablative strategies were adopted in addition to PVI. In these populations, there is indeed a reduction in procedural times in favor of LVA ablation compared to performing lines, boxes, and CFAE ablation. It should be noted, however, that after removing the VOLCANO trial [14] through leave-one-out analysis, a trend towards lower procedure time was found, despite not reaching statistical significance. This study involved a waiting time of at least 20 min after the last ablation to test the PVI, which might limit the difference among treatment groups. Finally, it should be considered that the patient-tailored ablation strategy based on the presence of LVA allows to avoid unnecessary ablations and thus to save time in patients diagnosed with PeAF but without LVA.

In addition, no difference in fluoroscopy time was demonstrated between the groups, also when excluding the VOLCANO trial [14] from the analysis: in this study, unlike all the others included, the majority of patients underwent PVI using cryoballoon, which involves a greater use of fluoroscopy.

### 4.5. Novelty of the Current Metanalysis and Clinical Implications

Pulmonary veins are a recognized trigger for AF initiation [4] and PVI is the cornerstone of AF ablation [5,11,26]. The prevailing consensus recognizes the substantial role of the arrhythmogenic substrate in the LA, contributing to the heightened recurrence rates seen in non-paroxysmal forms of AF [27]. Over recent years, numerous ablation strategies have been proposed to modify this substrate [28]. However, regrettably, none has yielded sufficient evidence to warrant widespread adoption in daily clinical practice. It has also been proposed to use cardiac magnetic resonance imaging, which can identify areas of fibrosis, to guide ablation. However, this approach has not shown any benefit [29] likely due to the current techniques’ limited ability to accurately identify fibrosis in such a thin wall. EAM, on the other hand, appears to be a promising method for identifying diseased areas of the LA. Although there are evidences from RCTs supporting LVA ablation in patients undergoing PVI, neither the guidelines [5,11] nor the recent EHRA expert consensus [29] provide clear recommendations on this matter. The findings of the current meta-analysis appear to support the advantages of ablating LVAs, as identified through electroanatomic voltage mapping, in addition to PVI. In contrast to prior meta-analyses on the topic [12,21,30,31,32], we exclusively focused on randomized studies and incorporated the most recent published trials, thereby substantially augmenting the number of patients analyzed and enhancing the overall statistical power of our analysis. Moreover, recognizing the clinical diversity inherent in studies on LVA, we explored this variability through numerous subgroup analyses.

A meta-analysis recently published by Rivera et al. [33] has shown results similar to the present meta-analysis. However, that study included ten trials, three more than ours. These trials were among the studies we extracted and excluded for various reasons. In the Hwang et al. [34] study, the target of the ablation was the CFAE within the LVA rather than directly the low voltages, which are instead the central focus of our meta-analysis. In the Kumagai et al. [35] study, patients with LVA were randomized to receive either posterior wall isolation (box) alone or box + LVA ablation; therefore, a subset of patients with LVA underwent posterior wall isolation without any data on the actual location of the LVAs in these patients. Furthermore, this study is aimed at measuring the effect of LVA ablation in addition to posterior wall isolation rather than comparing the two ablative techniques. Lastly, in the study by Wang et al. [36], all patients randomized to substrate modification ablation underwent a roof ablation line, followed by different ablative strategies depending on the extent of the LVAs, making this study very heterogeneous and unrelatable. For these reasons, we did not include these studies; we instead opted for those deemed more reliable and satisfying our inclusion and exclusion criteria. Furthermore, in this meta-analysis, unlike that of Rivera et al. [33], we analyzed the VOLCANO trial [14] considering the data from the extended 2-year follow-up.

### 4.6. Limitations

This meta-analysis has several limitations. First, six out of seven included studies randomized patients before voltage mapping was performed, resulting in a substantial portion of patients in the intervention arm without LVA. For this reason, we conducted a sensitivity analysis considering only patients with LVA. Second, there is variability among the studies regarding the type of diagnosis; some studies included patients with PeAF, while others included patients with PAF. Third, the ablation strategy and energy type used are not uniform across the studies. Lastly, some trials do not report raw data on atrial arrhythmia recurrences in the LVA population. For some of these studies, we derived the data from Kaplan-Meier curves, which may introduce errors in data extraction.

## 5. Conclusions

In AF patients, LVA ablation in addition to PVI reduces atrial arrhythmia recurrences without an increase in procedure time, fluoroscopy time, and complication rate.

## Figures and Tables

**Table 1:** Included studies characteristics. AF atrial fibrillation, PVI pulmonary vein isolation, LVA low-voltage area.

Study	Design	Population	Experimental Arm	Control Arm	Energy Source	Endpoint	Follow Up
B. Yang et al. [16] (STABLE-SR)	Multicenter, single-blinded, randomized clinical trial	Non-paroxysmal AF	PVI + cavotricuspid isthmus ablation + LVA ablation	PVI + STEPWISE ablation	Radiofrequency	Freedom from documented atrial arrhythmias for >30 s without the use of antiarrhythmic drugs	18 months
G. Yang et al. [17] (STABLE-SR II)	Multicenter, single-blinded, randomized clinical trial	Non-paroxysmal AF	PVI + LVA ablation	PVI	Radiofrequency	Freedom from documented atrial arrhythmias for >30 s without the use of antiarrhythmic drugs	18 months
H. Chen et al. [15] (STABLE-SR III)	Multicenter, single-blind, randomized clinical trial	Paroxysmal AF, 65 to 80 years	PVI + LVA ablation	PVI	Radiofrequency	Freedom from documented atrial arrhythmias for >30 s without the use of antiarrhythmic drugs	23 months
Y. Huo et al. [19] (ERASE)	Multicenter, single-blinded, randomized clinical trial	Persistent AF	PVI + LVA ablation	PVI	Radiofrequency	Freedom from documented atrial arrhythmias for >30 s	12 months
S. Kircher et al. [20]	Single-center, single-blinded, randomized clinical trial	Paroxysmal and Persistent AF	PVI + LVA ablation	PVI (+ posterior wall box and posterior mitral annulus line in persistent AF)	Radiofrequency	Freedom from documented atrial arrhythmias for >30 s	12 months
B. Kaiser et al. [18]	Single-center, single-blinded, randomized clinical trial.	Persistent AF	PVI + LVA ablation	PVI + linear lesions + CFAE ablation	Radiofrequency	Freedom from any atrial arrhythmia after the 90 days blanking period without the use of antiarrhythmic drugs	12 months
M. Masuda et al. [23] (VOLCANO)	Multicenter, single-blinded, randomized clinical trial.	Paroxysmal AF	PVI + LVA ablation	PVI alone	Radiofrequency or Cryoballoon	Freedom from any atrial arrhythmia after the 90 days blanking period without the use of antiarrhythmic drugs	24 months

**Table 2:** Patient characteristics. Means are presented ±SD or median (IQR). LVA low-voltage area, AF atrial fibrillation, LVEF left ventricle ejection fraction, LAD left atrium diameter.

Study		Patients, No.	Age, Mean	Male, %	AF Duration (Months), Mean	CHA2DS2-VASc Score, Mean or Median	LVEF, %	LAD, mm
STABLE-SR [16]	LVA ablation	114	57.1 ± 9.5	81	18.9 ± 29	-	61.8 ± 7.7	41.1 ± 5.3
	Non-LVA ablation	115	57.6 ± 8.4	74	15.9 ± 33	-	62.0 ± 6.6	40.7 ± 4.8
STABLE-SR II [17]	LVA ablation	134	60.6 ± 9.4	67	6.0 (2.0–15.5)	-	61.3 ± 9.2	41.4 ± 5.9
	Non-LVA ablation	142	60.4 ± 9.6	70	6.0 (1.0–12.0)	-	62.1 ± 6.8	42.5 ± 5.3
STABLE-SR III [15]	LVA ablation	219	70.2 ± 4.7	51	24.0 (6.0–48.0)	2.3 ± 0.8	62.4 ± 5.3	38.8 ± 5.4
	Non-LVA ablation	219	70.7 ± 4.1	49	14 (4.0–48.0)	2.5 ± 1.0	62.4 ± 5.4	38.8 ± 5.4
ERASE [19]	LVA ablation	161	65 ± 10	70	31 (8–77)	3 (2–4)	53 ± 12	45 ± 7
	Non-LVA ablation	163	66 ± 10	64	31 (12–77)	3 (2–4)	54 ± 11	45 ± 6
Kircher et al. [20]	LVA ablation	62	62 ± 10	58	54 (24–87)	2 (1–3)	59 ± 9	43 ± 6
	Non-LVA ablation	62	63 ± 9	66	60 (36–111)	2 (1–3)	61 ± 7	42 ± 6
Kaiser et al. [18]	LVA ablation	50	65.20 ± 8.94	66	-	2.67 ± 1.58	53.35 ± 5.23	-
	Non-LVA ablation	50	67.53 ± 10.83	72	-	2.55 ± 1.74	50.29 ± 9.61	-
VOLCANO [23]	LVA ablation	30	75.3 ± 7.2	30	4 (2–14)	3.6 ± 1.2	64 ± 14	40 ± 6
	Non-LVA ablation	32	74.7 ± 8.0	28	5 (2–23)	3.3 ± 1.3	65 ± 10	38 ± 5

## Supplementary Materials

The following supporting information can be downloaded at: https://www.mdpi.com/article/10.3390/jcm13154541/s1, Figure S1: Quality assessment adapted from Cochrane’s Collaboration Tool (RoB2) for randomized controlled trials; Figure S2: Funnel plot for publication bias; Figure S3: Forrest plot of atrial arrhythmia recurrence (fixed analysis); Figure S4: Results of leave-one-out method in sensitivity analysis for procedure time; Figure S5: Results of leave-one-out method in sensitivity analysis for fluoroscopy time; Figure S6: Results of leave-one-out method in sensitivity analysis for complications; Figure S7: Forrest plot of subgroup analysis for the primary outcome based on type of atrial fibrillation; Figure S8: Forrest plot of subgroup analysis for the primary outcome based on multicentric vs monocentric trials; Figure S9: Forrest plot of subgroup analysis for the primary outcome based on ROB2 assessment; Figure S10: Forrest plot of subgroup analysis for the primary outcome based on ablation strategy in control group; Figure S11: Forrest plot of subgroup analysis for the secondary outcome procedure time based on type of AF; Figure S12: Forrest plot of subgroup analysis for the secondary outcome procedure time based on ablation strategy in control group. Table S1: Metaregression for the primary outcome.

## Abbreviations

AF	Atrial Fibrillation
CFAE	Complex Fractionated Atrial Electrogram
EAM	Electroanatomical Mapping
LAD	Left Atrium Diameter
LVEF	Left Ventricular Ejection Fraction
LVA	Low-Voltage Area
MD	Mean Difference
MRI	Magnetic Resonance Imaging
OR	Odds Rratio
PAF	Paroxysmal Atrial Fibrillation
PeAF	Persistent Atrial Fibrillation
PRISMA	Preferred Reporting Items for Systematic Reviews and Meta-Analyses
PROSPERO	International Prospective Register of Systematic Reviews
PVI	Pulmonary Vein Isolation
RCTs	Randomized Clinical Trials
REML	Restricted Maximum Likelihood
ROB2	Cochrane Risk of Bias Tool
