# Supplementary material for: Low-Voltage Area Ablation in Addition to Pulmonary Vein Isolation in Patients with Atrial Fibrillation: A Systematic Review and Meta-Analysis
Source: J Clin Med. 2024 Aug 3;13(15):4541. doi: 10.3390/jcm13154541 (PMC11313645; doi:10.3390/jcm13154541)
Supplement: Supplementary file 1 [file jcm-13-04541-s001.zip › SUPPLEMENTARY_META.pdf]

SUPPLEMENTARY MATERIALS

Supplementary Figure 1. Quality assessment adapted from Cochrane's Collaboration Tool (RoB2) for randomized controlled trials.

	D1	D2	D3	D4	D5	Overall
STABLE-SRIII	+	+	+	+	+	+
STABLE-SR	+	+	+	+	+	+
Kaiser et al.	!	!	+	-	+	!
STABLE-SRII	+	+	+	+	+	+
ERASE	+	+	+	+	+	+
Kircher et al.	+	!	+	!	+	!
VOLCANO	+	+	+	!	+	!

D1: Randomisation process

D2: Deviations from the intended interventions

D3: Missing outcome data

D4: Measurement of the outcome

D5: Selection of the reported result

Supplementary Figure 2. Funnel plot for publication bias. CI confidence interval.

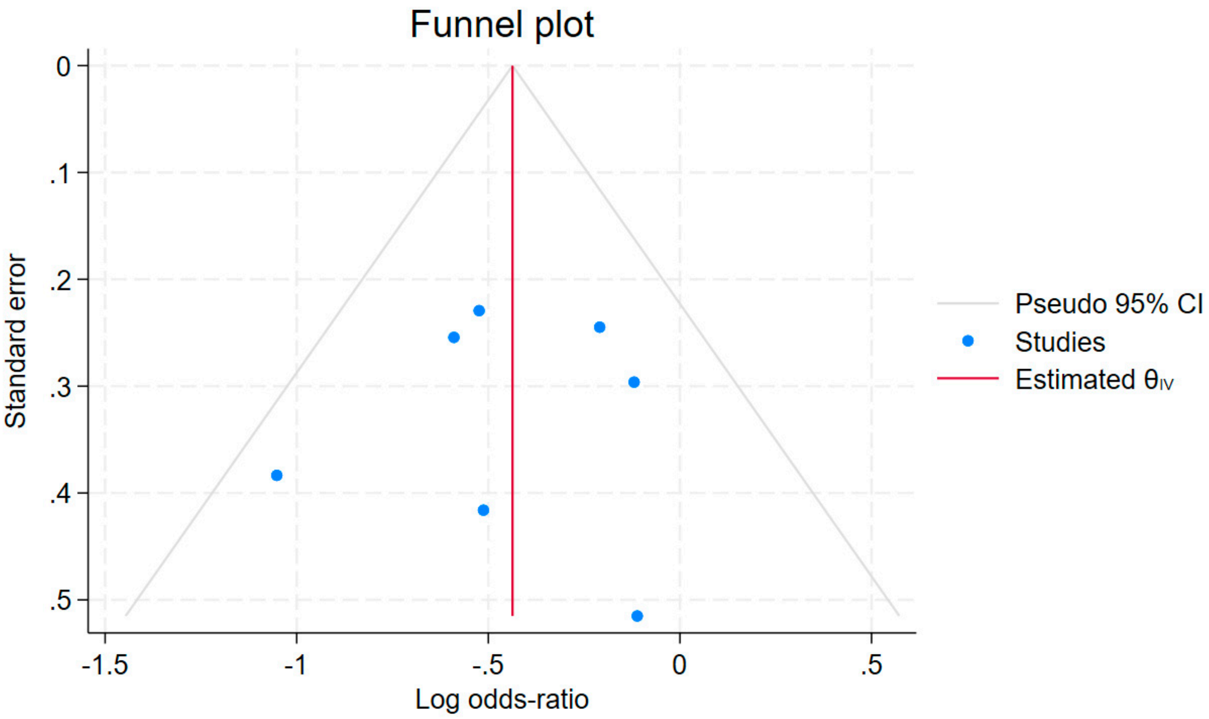

Supplementary Figure 3. Forrest plot of atrial arrhythmia recurrence (fixed analysis).

LVA low-voltage ablation, OR odds ratio, CI confidence interval.

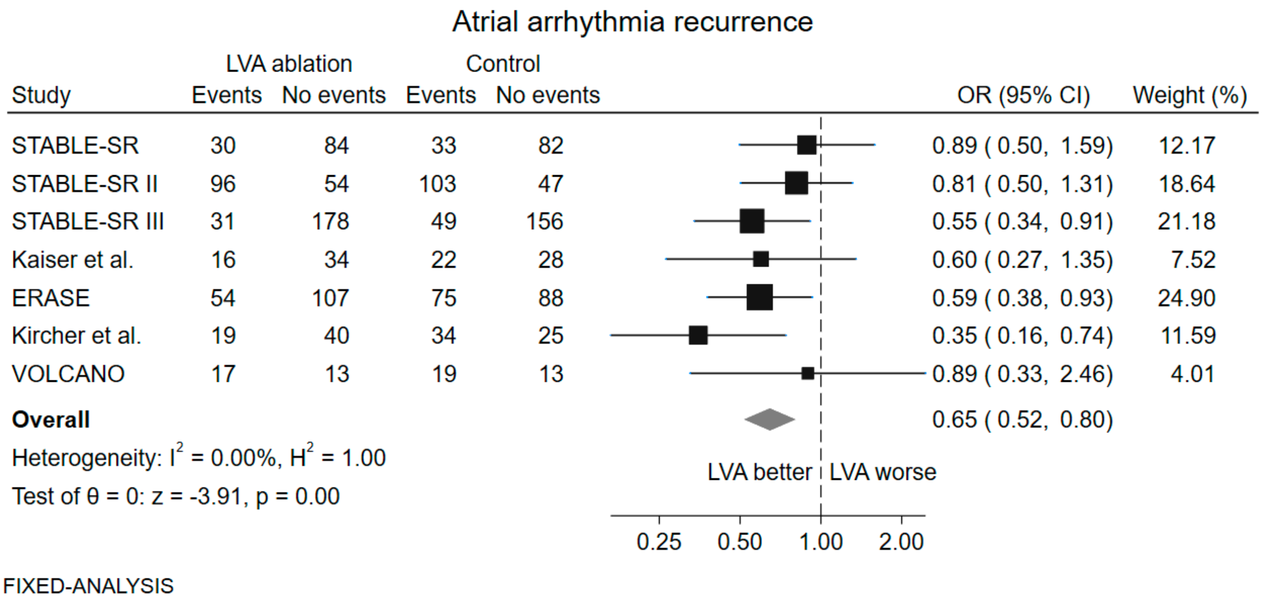

Supplementary Figure 4. Results of leave-one-out method in sensitivity analysis for procedure time.

MD mean difference, CI confidence interval.

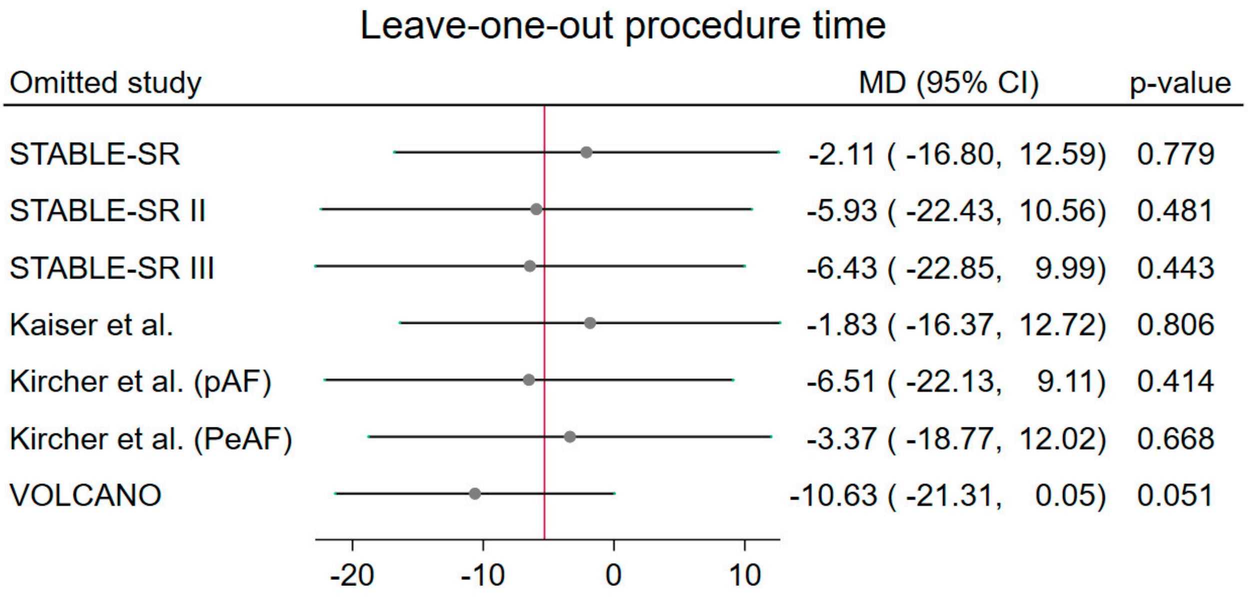

Supplementary Figure 5. Results of leave-one-out method in sensitivity analysis for fluoroscopy time.

MD mean difference, CI confidence interval.

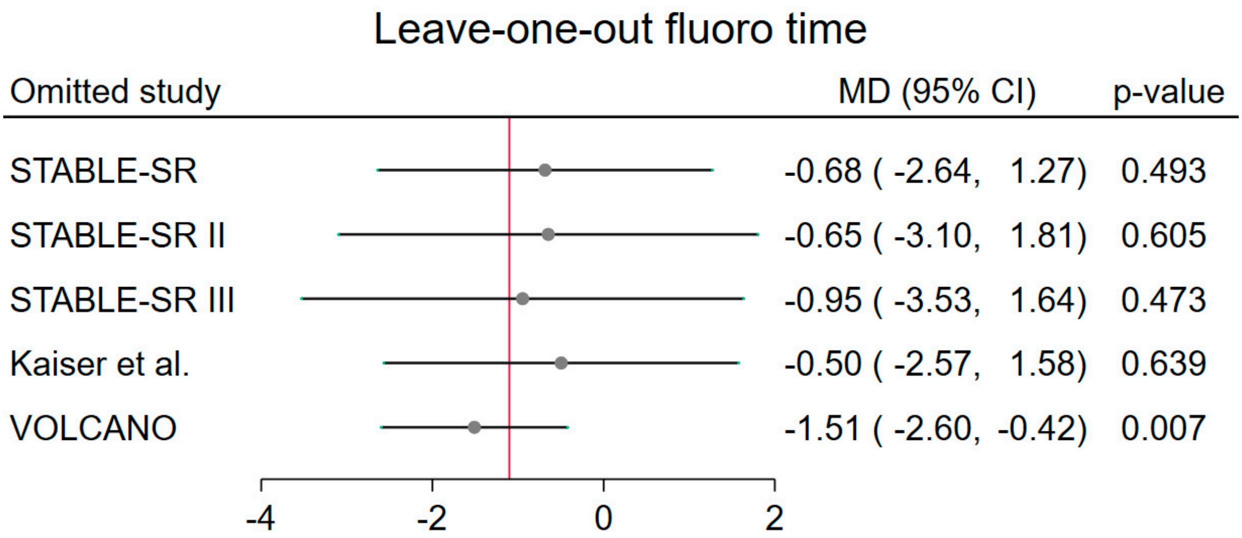

Supplementary Figure 6. Results of leave-one-out method in sensitivity analysis for complications.

MD mean difference, CI confidence interval.

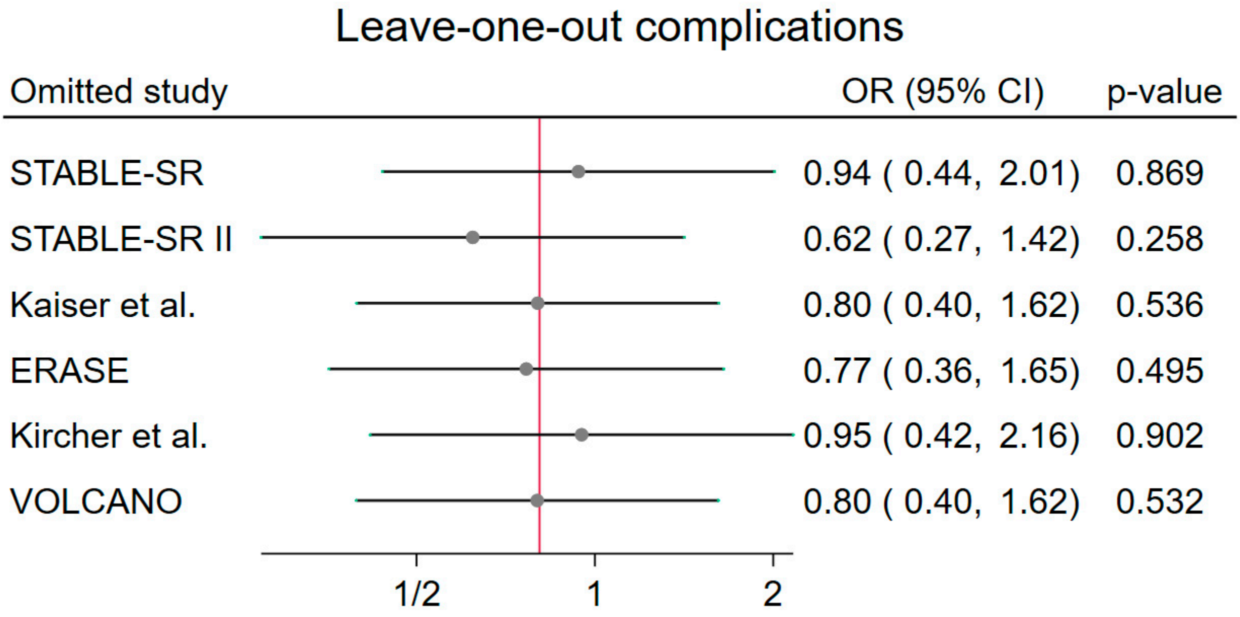

Supplementary Figure 7. Forrest plot of subgroup analysis for the primary outcome based on type of atrial fibrillation.

LVA low-voltage area, OR odds ratio, CI confidence interval.

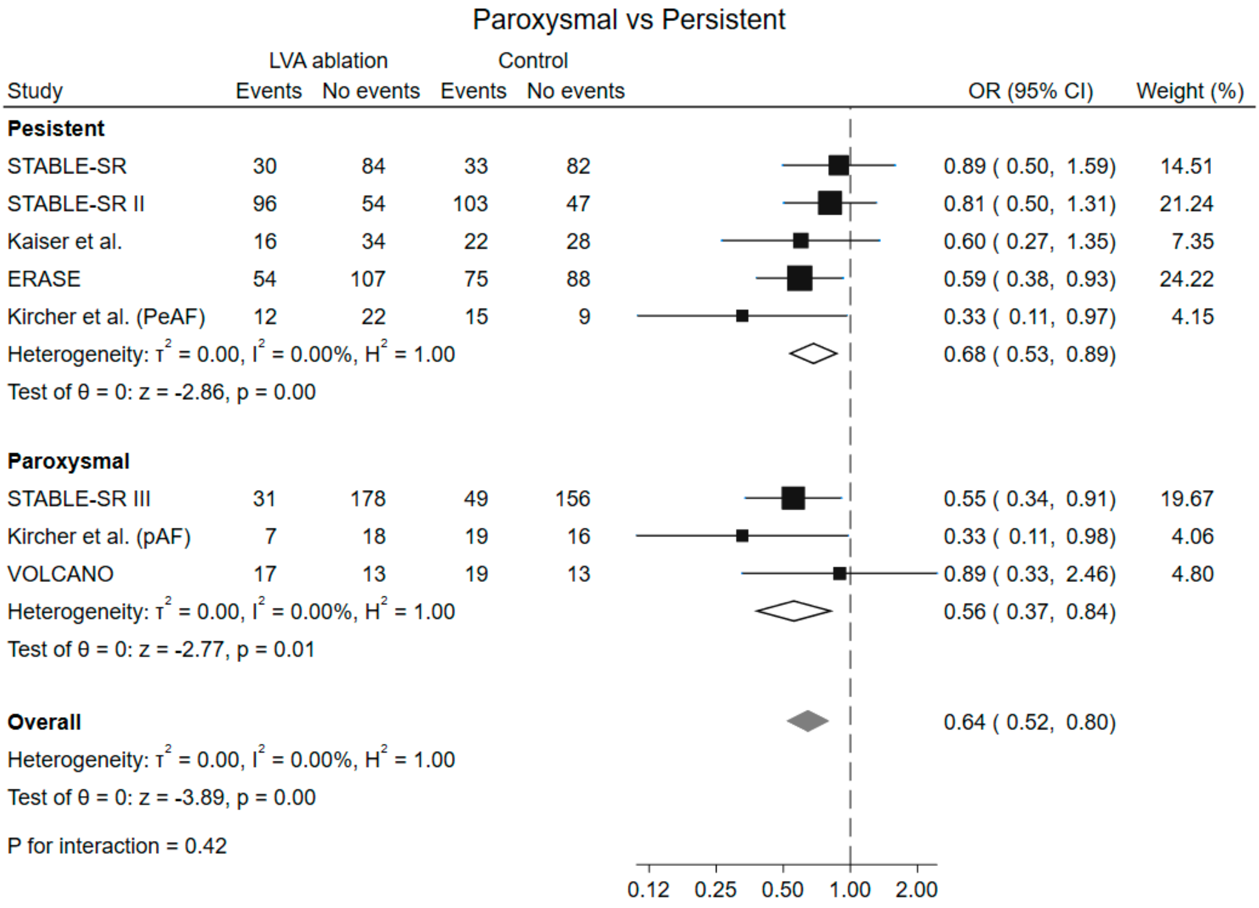

Supplementary Figure 8. Forrest plot of subgroup analysis for the primary outcome based on multicentric vs monocentric trials.

LVA low-voltage area, OR odds ratio, CI confidence interval.

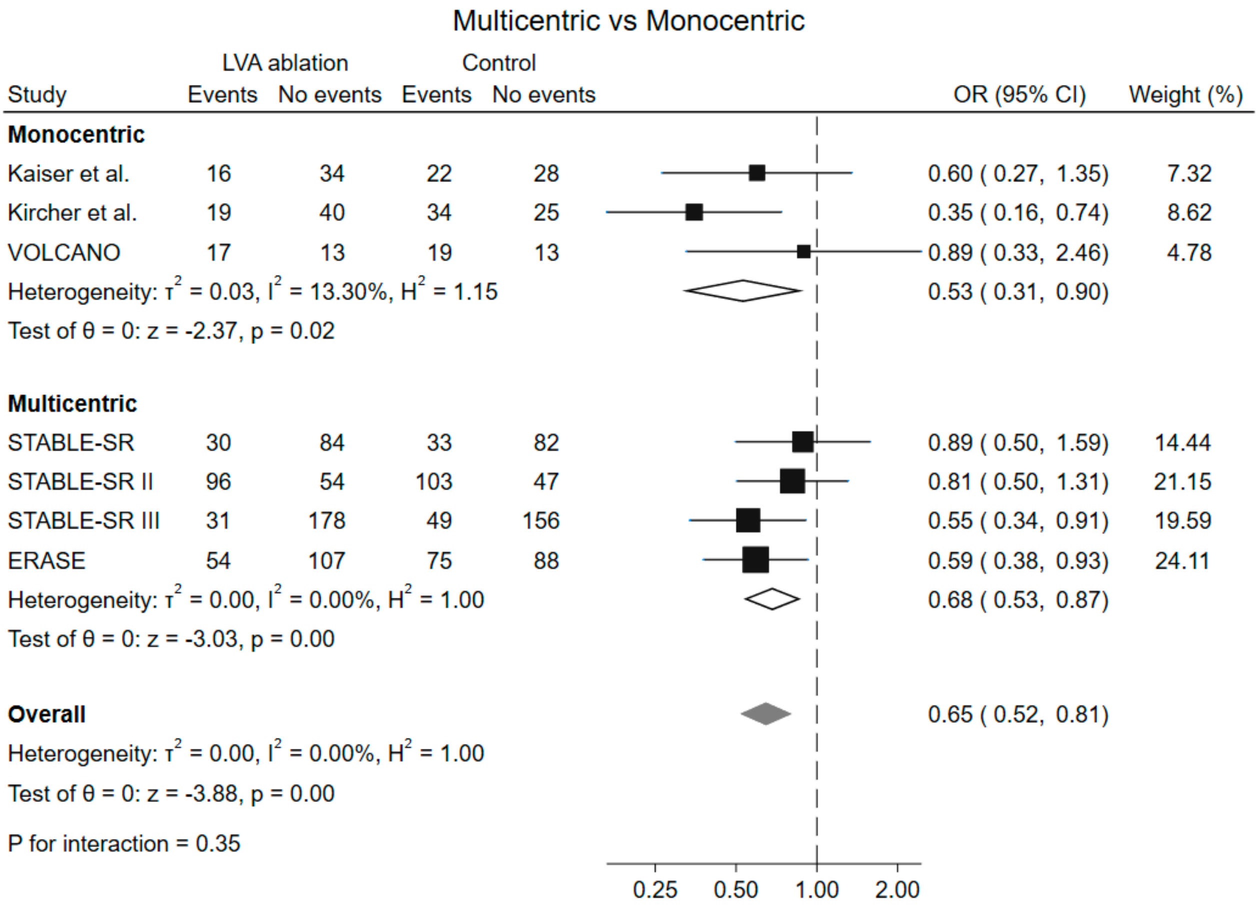

Supplementary Figure 9. Forrest plot of subgroup analysis for the primary outcome based on ROB2 assessment.

LVA low-voltage area, OR odds ratio, CI confidence interval.

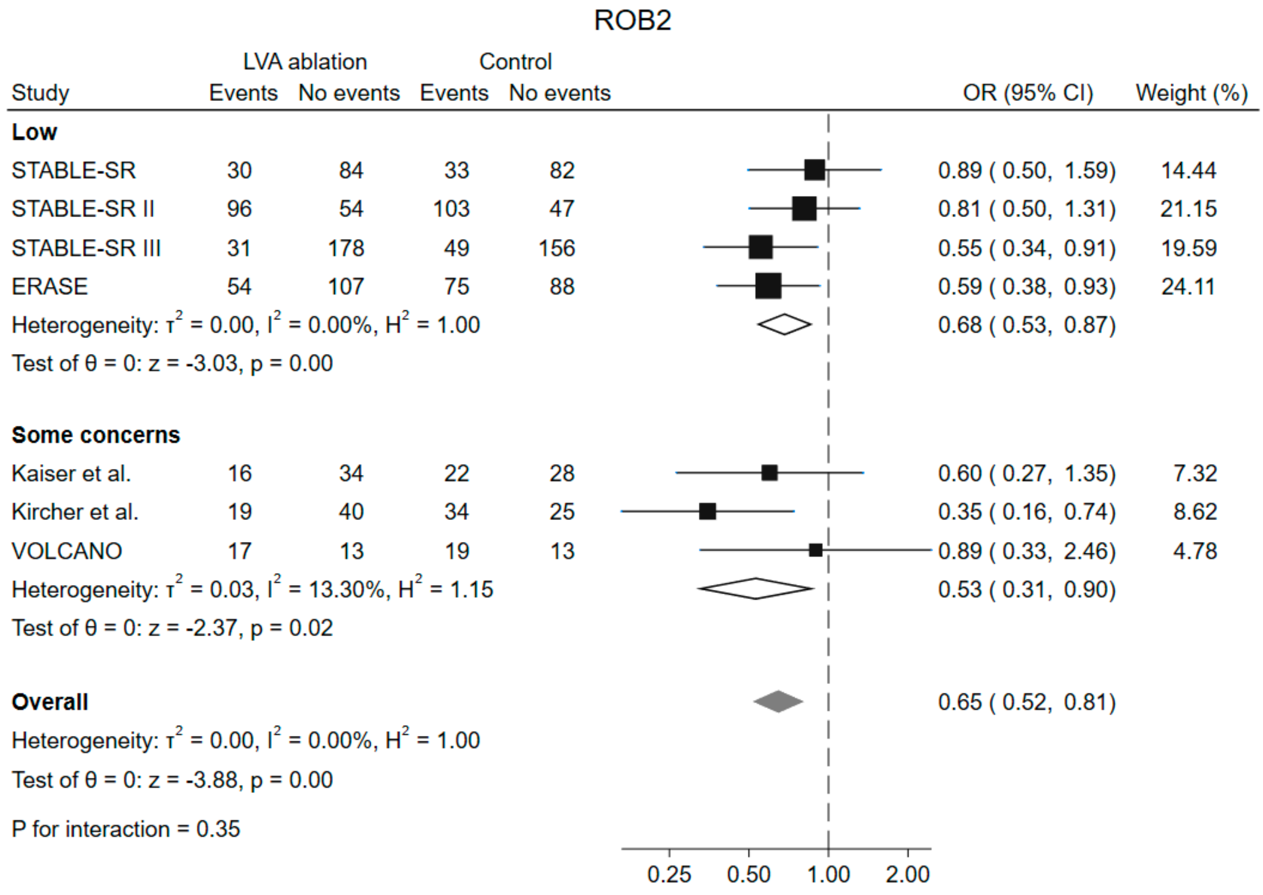

Supplementary Figure 10. Forrest plot of subgroup analysis for the primary outcome based on ablation strategy in control group.

LVA low-voltage area, OR odds ratio, CI confidence interval.

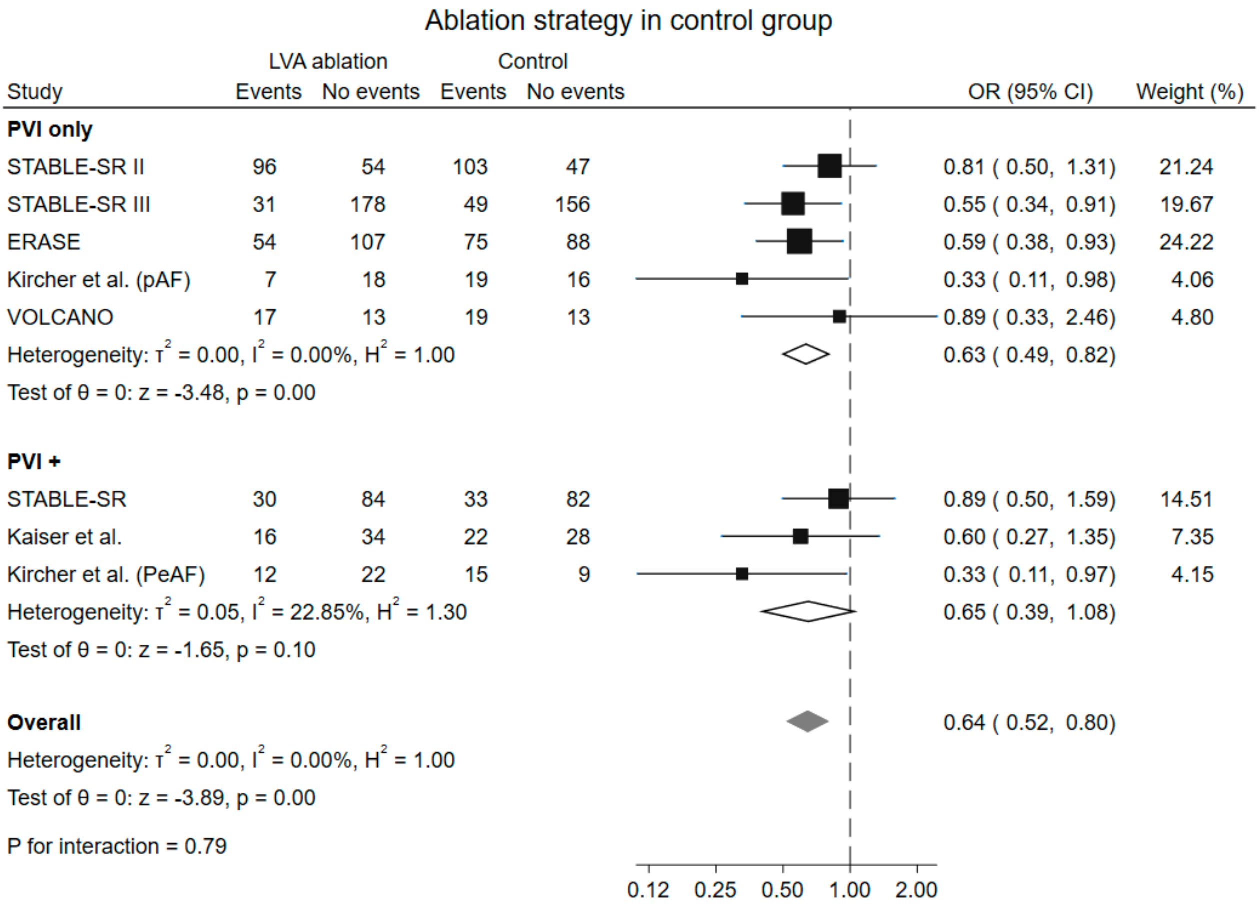

Supplementary Figure 11. Forrest plot of subgroup analysis for the secondary outcome procedure time based on type of AF. LVA low-voltage area, MD mean difference, CI confidence interval.

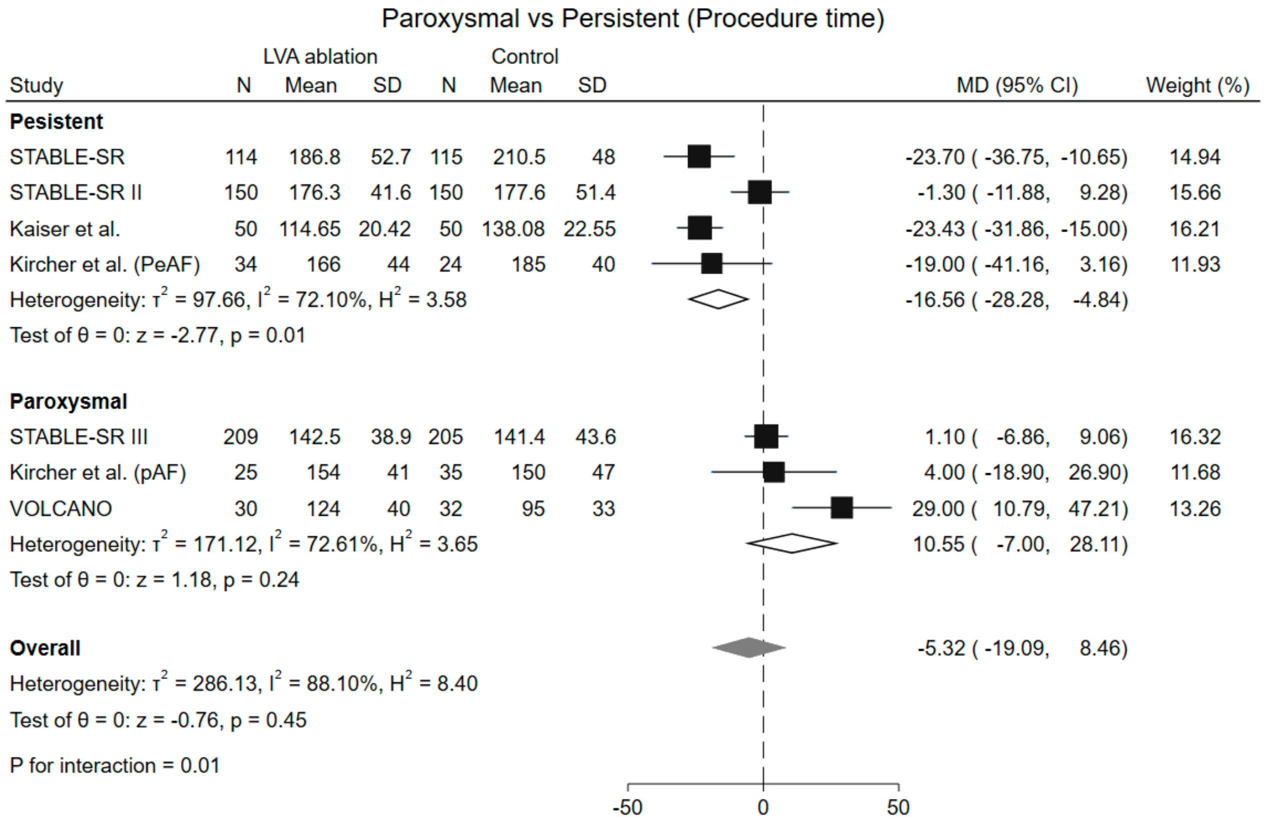

Supplementary Figure 12. Forrest plot of subgroup analysis for the secondary outcome procedure time based on ablation strategy in control group. LVA low-voltage area, MD mean difference, CI confidence interval.

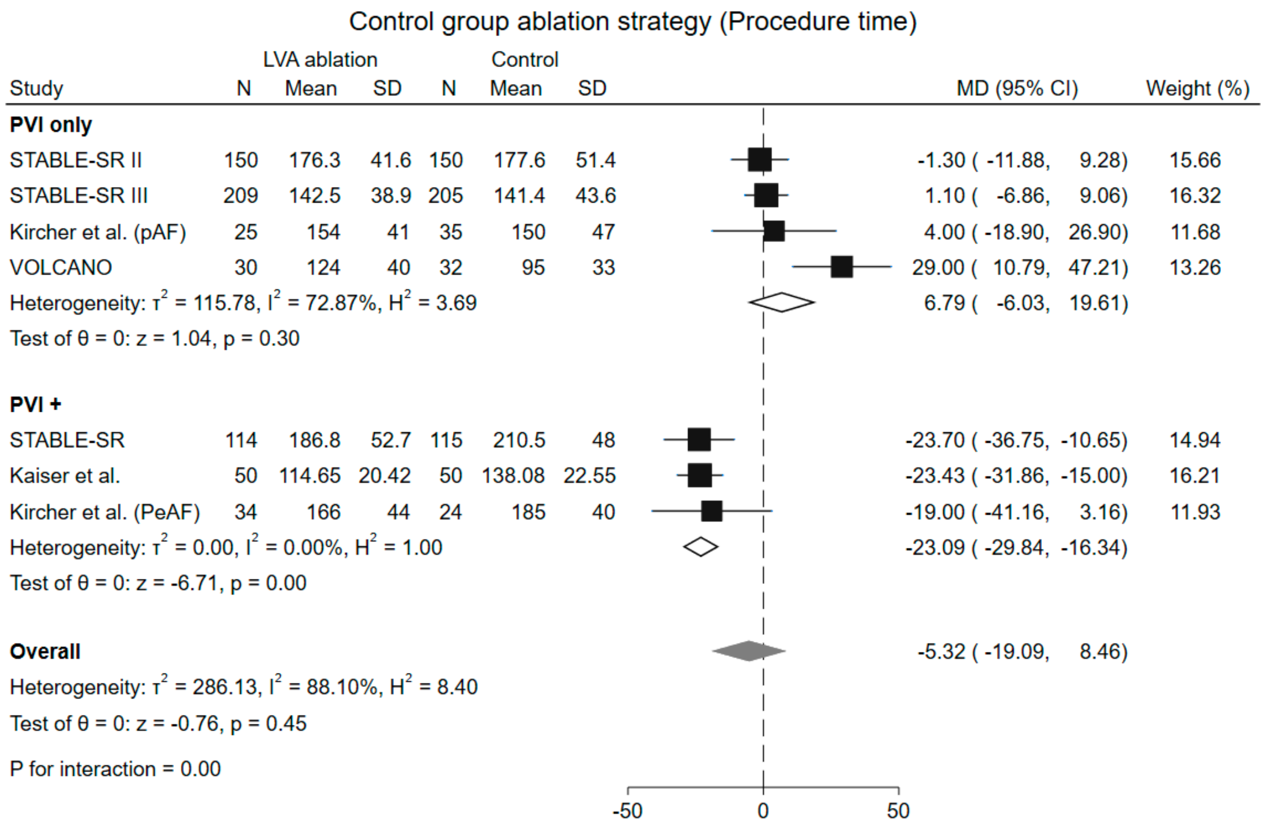

Supplementary Table 1. Metaregression for the primary outcome. LVEF left ventricle ejection fraction, LAD left atrium diameter, AF atrial fibrillation, CI confidence interval.

**Variable**	**Atrial Arrhythmia recurrence**			
	Coefficient	Lower 95% CI	Upper 95% CI	p-value
Age, year	-0.020	-0.064	0.025	0.385
Male	0.0005	-0.003	0.004	0.793
LVEF, %	0.019	-0.032	0.070	0.464
LAD, mm	-0.024	-0.145	0.098	0.703
CHA_2DS_2-VASc score	-0.140	-0.389	0.110	0.272
AF duration, months	-0.016	-0.032	-0.0005	0.043
