# Supplementary figures and images for: Low-Voltage Area Ablation in Addition to Pulmonary Vein Isolation in Patients with Atrial Fibrillation: A Systematic Review and Meta-Analysis
Source: J Clin Med. 2024 Aug 3;13(15):4541. doi: 10.3390/jcm13154541 (PMC11313645; doi:10.3390/jcm13154541)

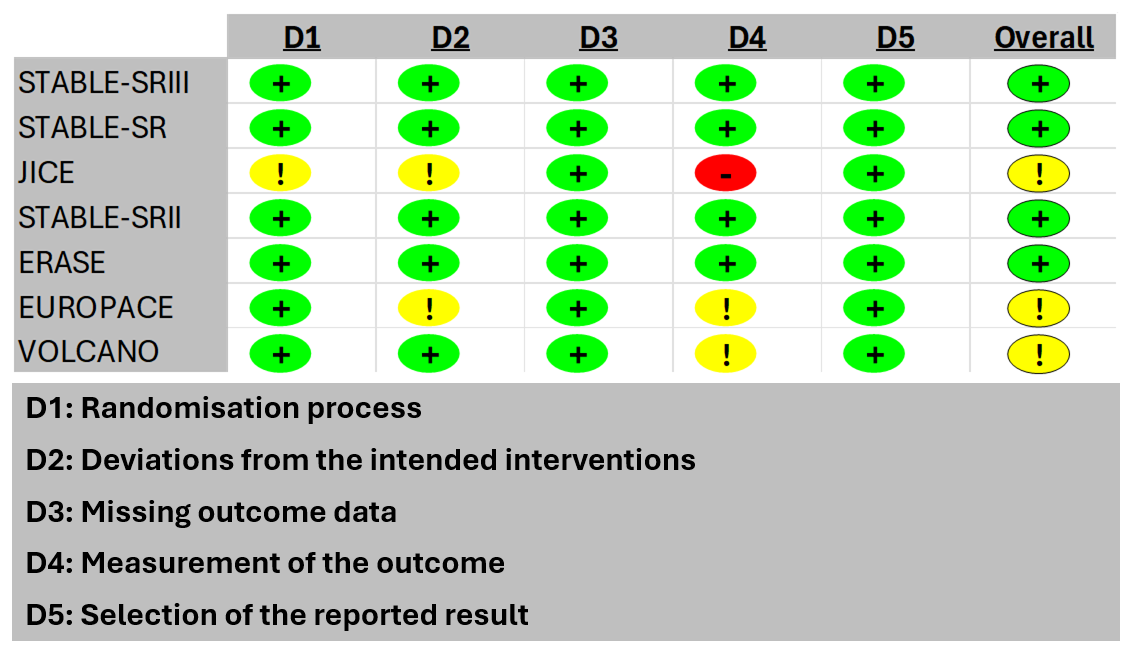

Supplement: Supplementary file 1 [file jcm-13-04541-s001.zip › Supplementary_Figure_1.PNG]

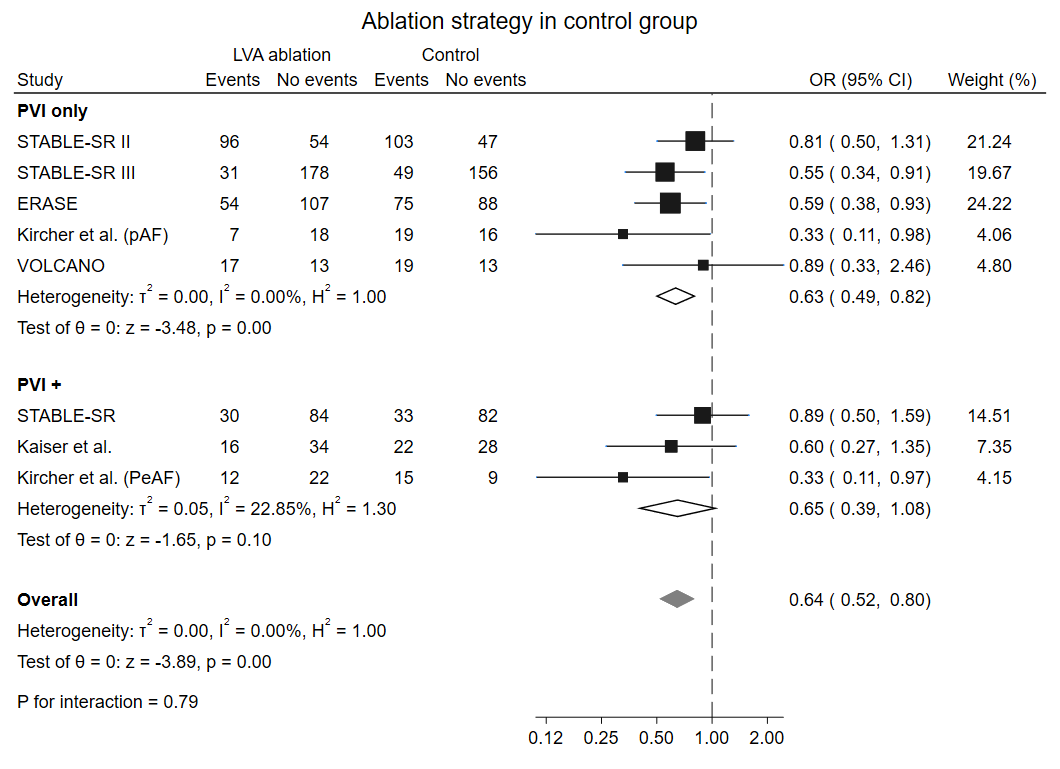

Supplement: Supplementary file 1 [file jcm-13-04541-s001.zip › Supplementary_Figure_10.png]

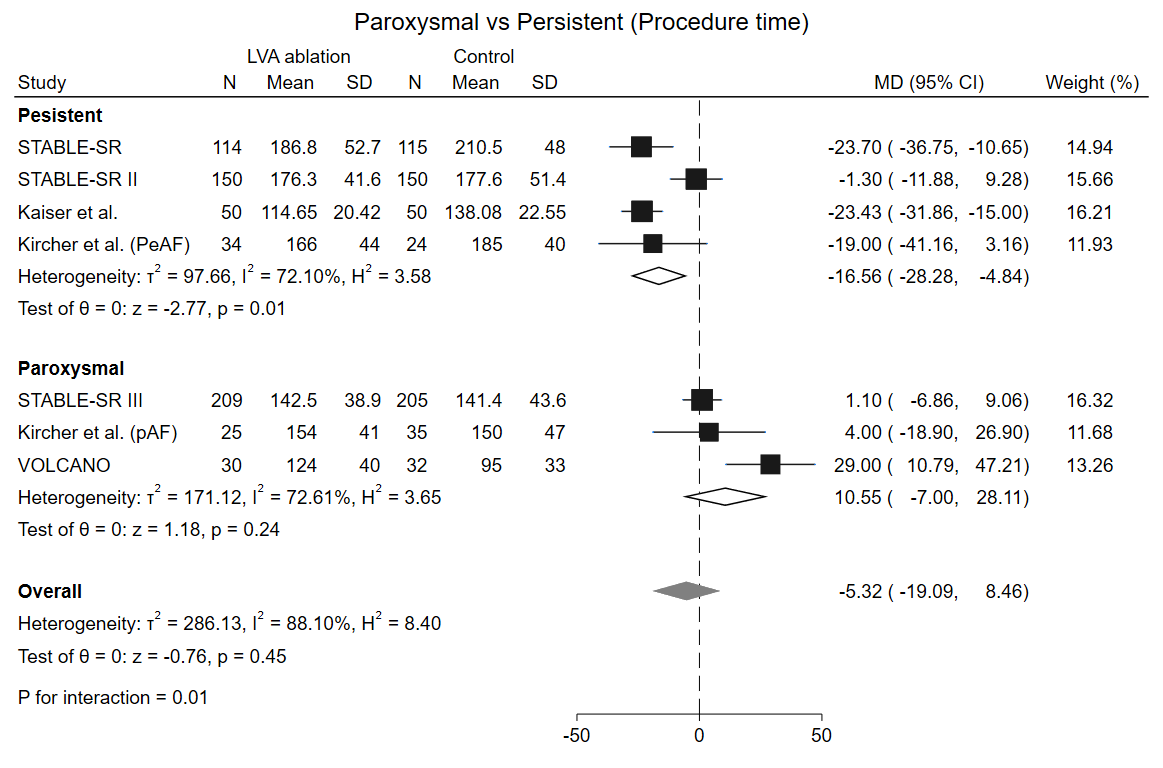

Supplement: Supplementary file 1 [file jcm-13-04541-s001.zip › Supplementary_Figure_11.png]

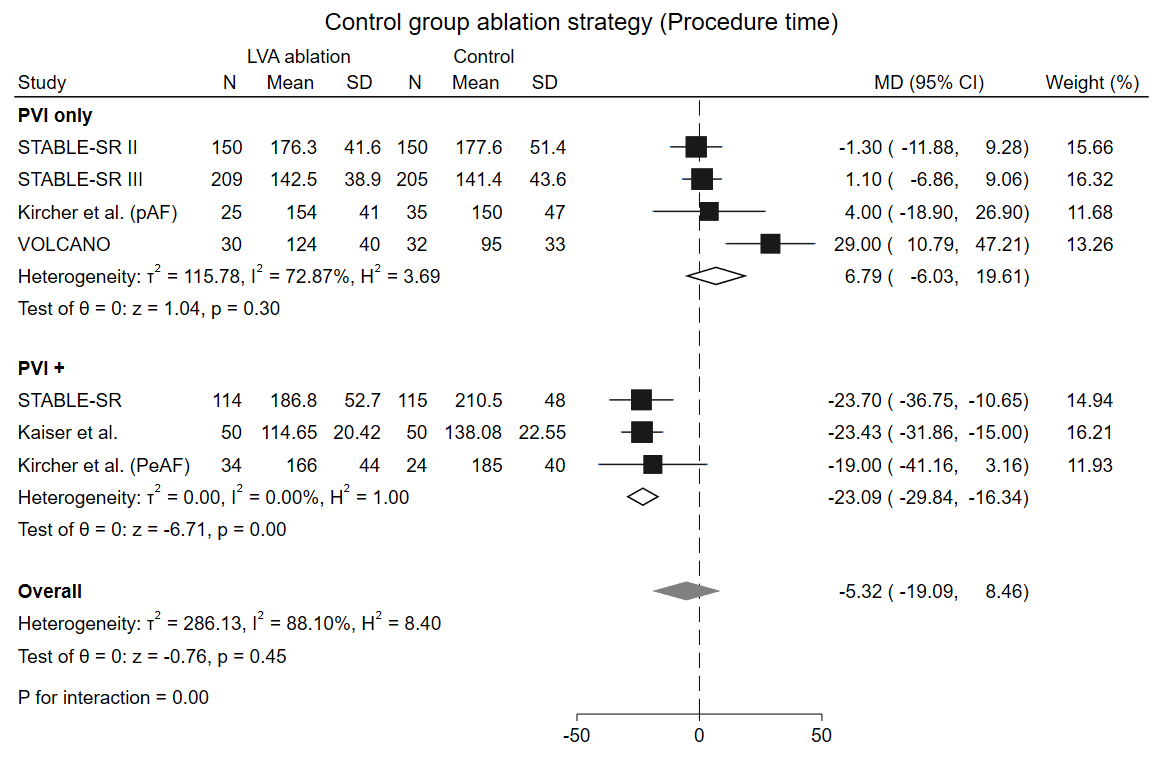

Supplement: Supplementary file 1 [file jcm-13-04541-s001.zip › Supplementary_Figure_12.png]

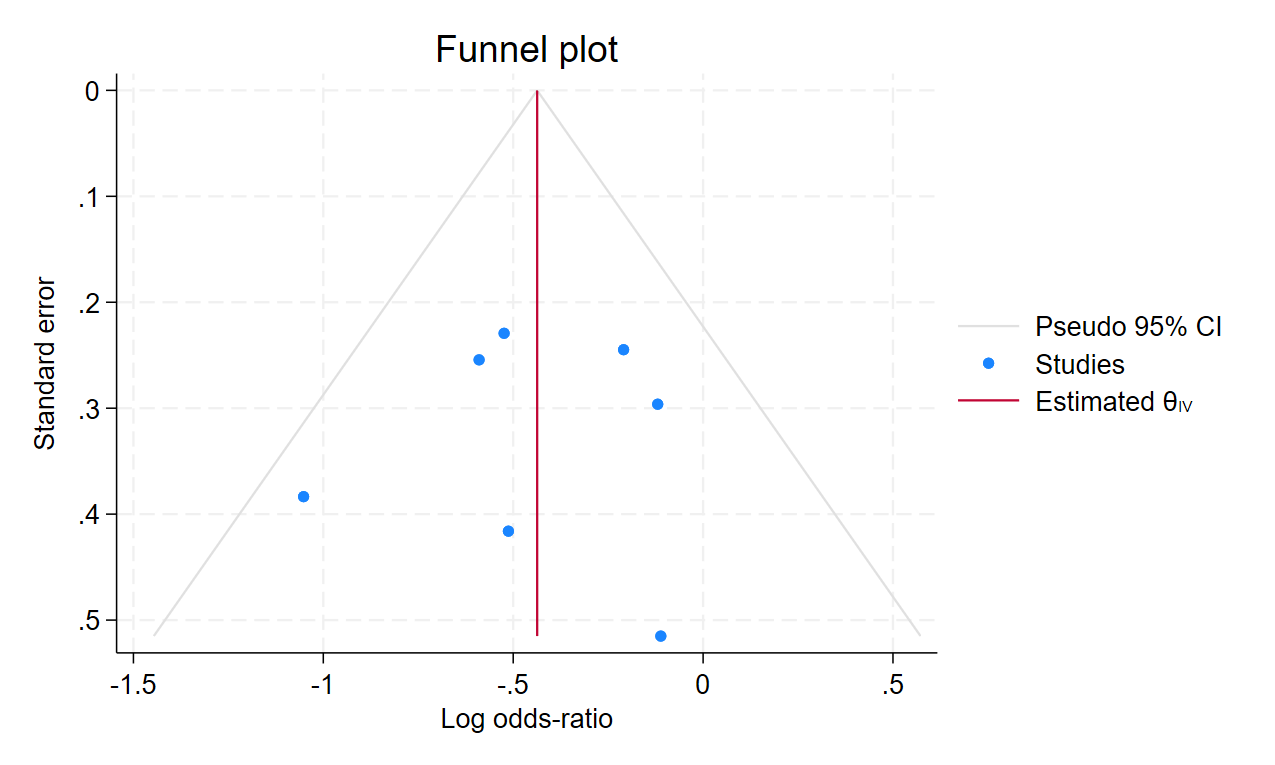

Supplement: Supplementary file 1 [file jcm-13-04541-s001.zip › Supplementary_Figure_2.png]

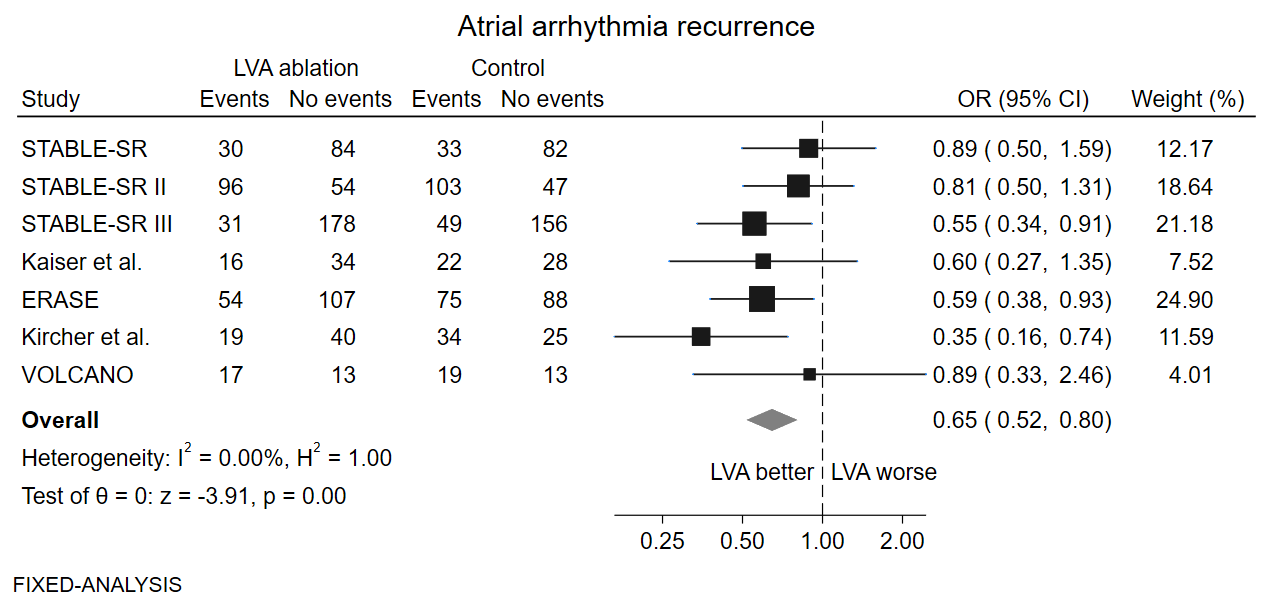

Supplement: Supplementary file 1 [file jcm-13-04541-s001.zip › Supplementary_Figure_3.png]

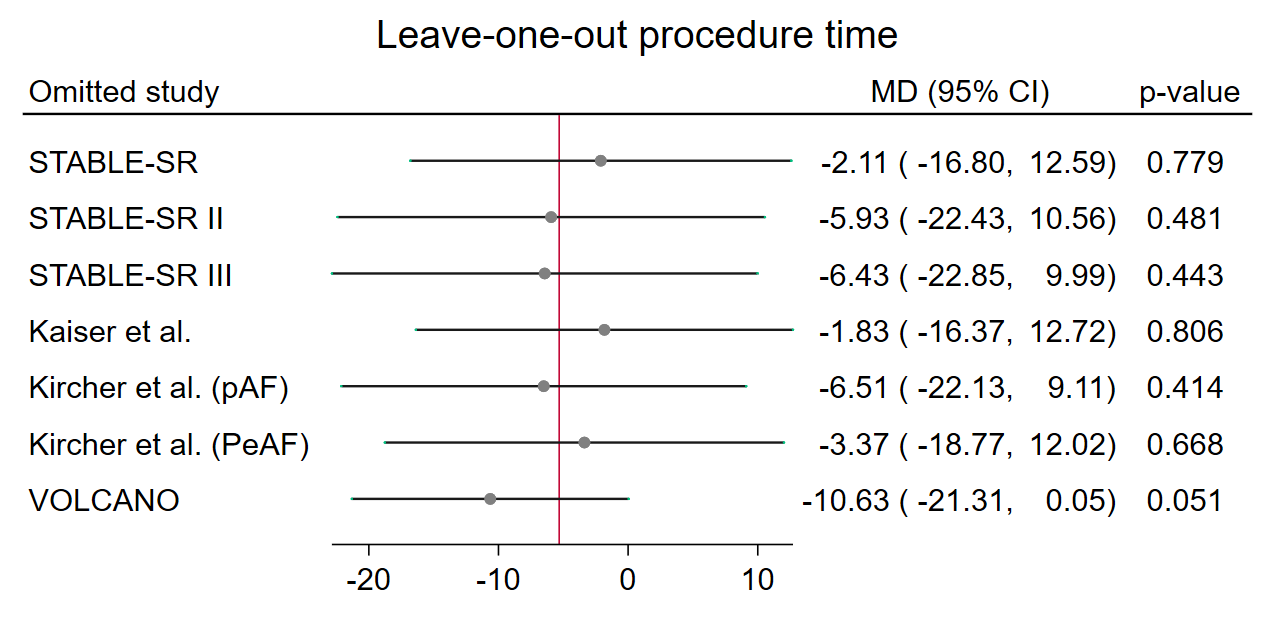

Supplement: Supplementary file 1 [file jcm-13-04541-s001.zip › Supplementary_Figure_4.png]

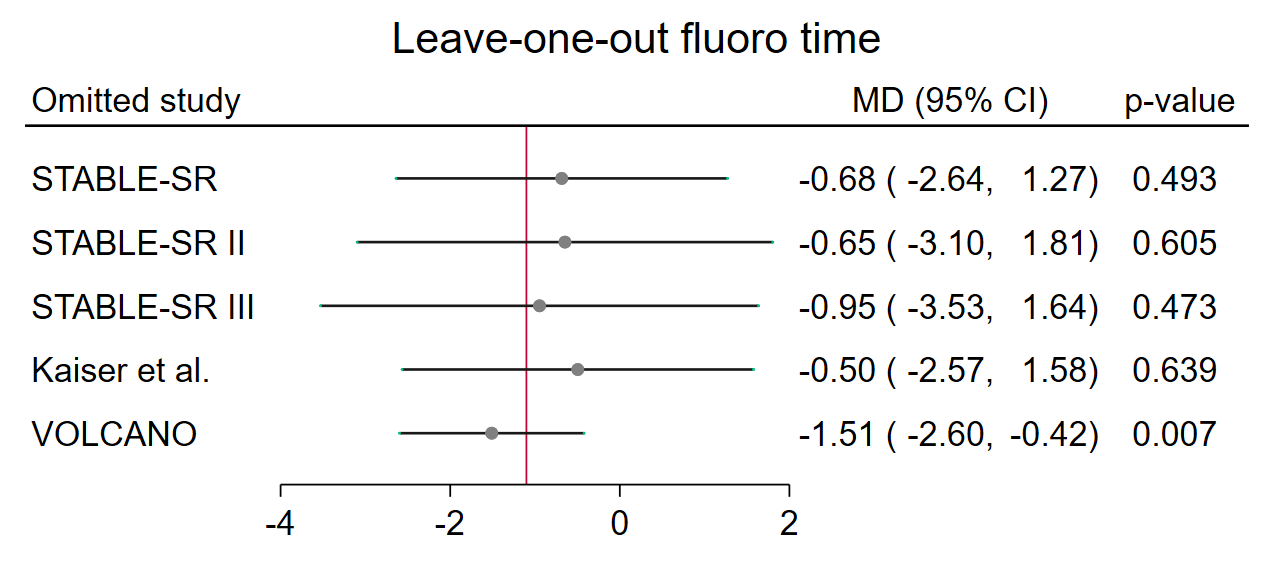

Supplement: Supplementary file 1 [file jcm-13-04541-s001.zip › Supplementary_Figure_5.png]

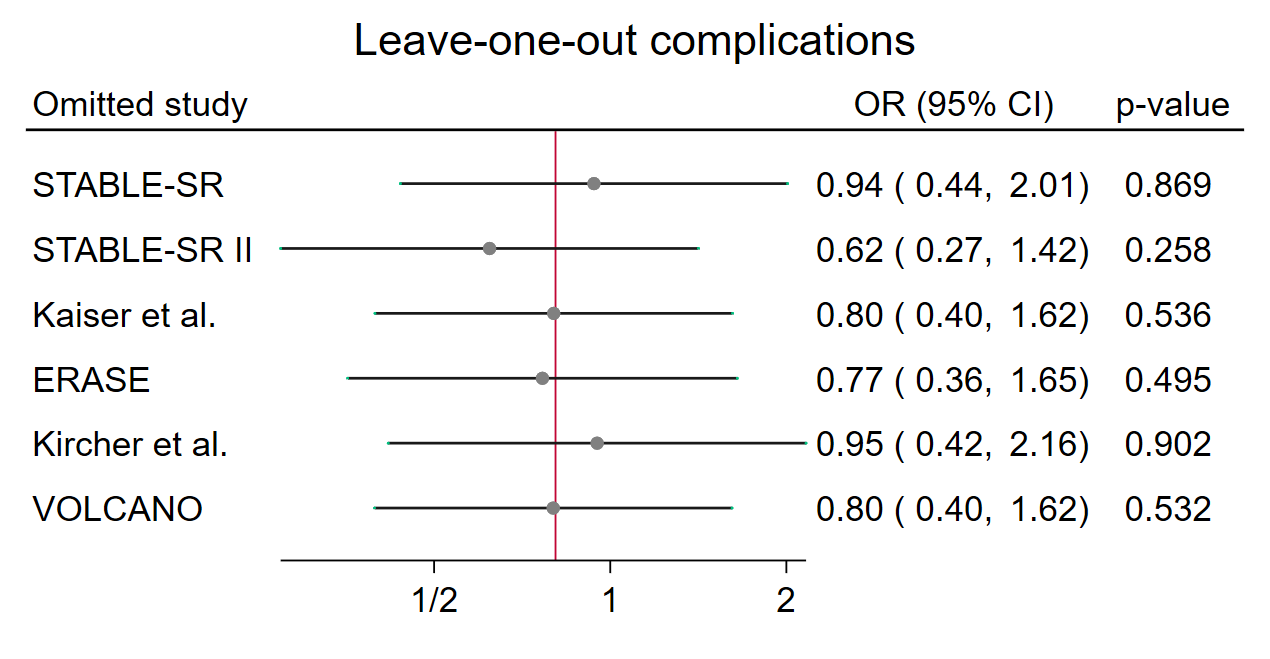

Supplement: Supplementary file 1 [file jcm-13-04541-s001.zip › Supplementary_Figure_6.png]

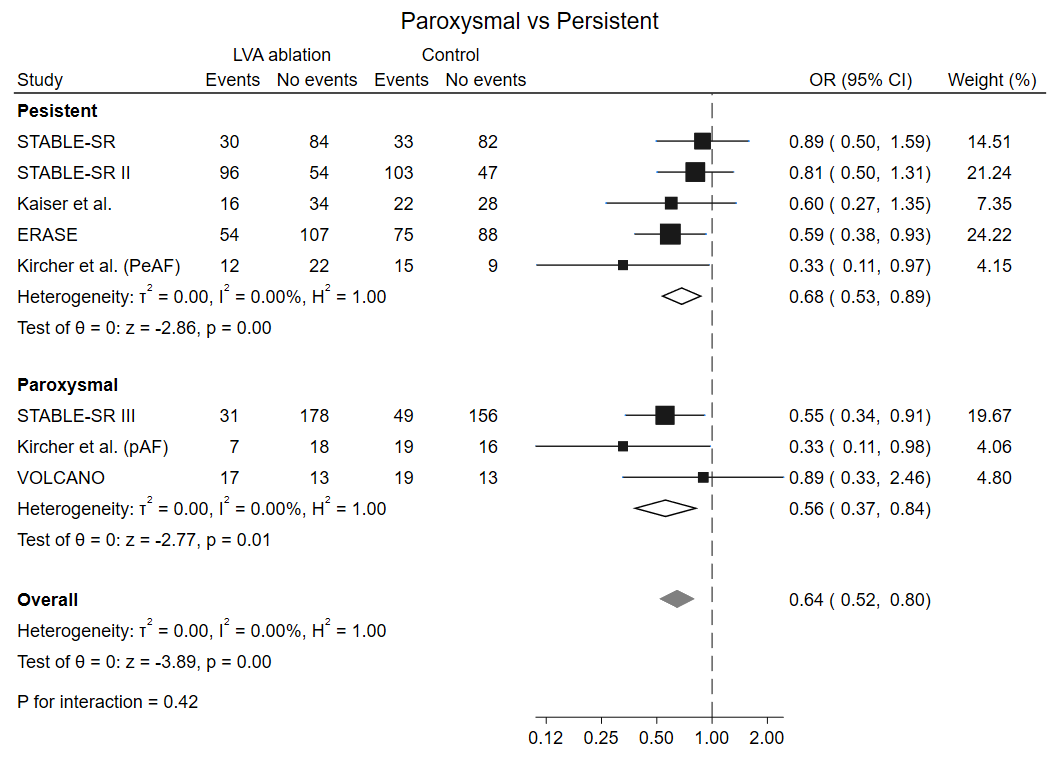

Supplement: Supplementary file 1 [file jcm-13-04541-s001.zip › Supplementary_Figure_7.png]

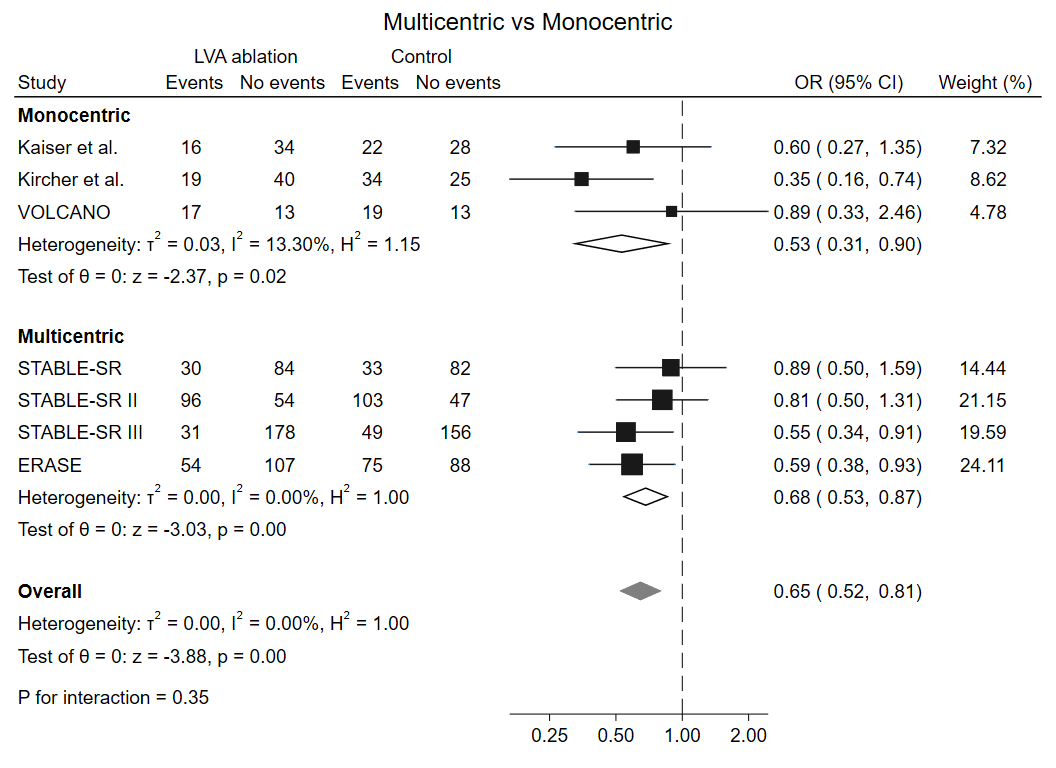

Supplement: Supplementary file 1 [file jcm-13-04541-s001.zip › Supplementary_Figure_8.png]

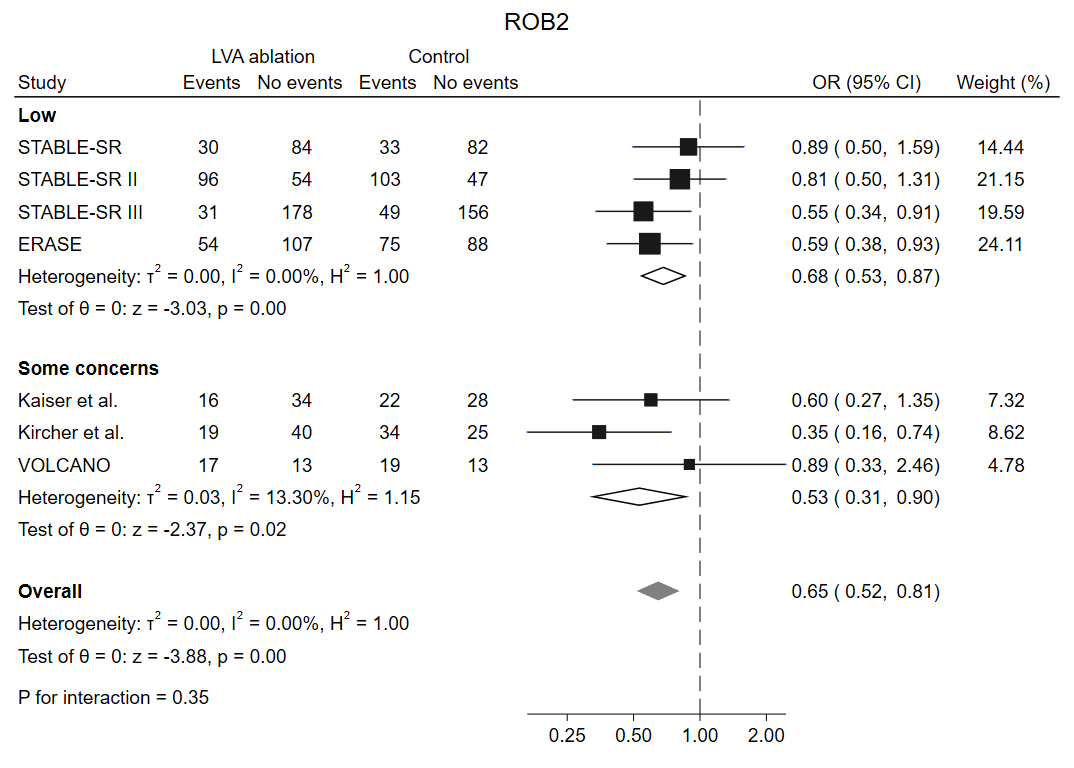

Supplement: Supplementary file 1 [file jcm-13-04541-s001.zip › Supplementary_Figure_9.png]
